# Impaired sleep affects quality of life in children during maintenance treatment for acute lymphoblastic leukemia: an exploratory study

**DOI:** 10.1186/1477-7525-9-25

**Published:** 2011-04-18

**Authors:** Raphaële RL van Litsenburg, Jaap Huisman, Peter M Hoogerbrugge, R Maarten Egeler, Gertjan JL Kaspers, Reinoud JBJ Gemke

**Affiliations:** 1 Department of pediatrics, VU University Medical Center, Amsterdam, Netherlands; 2 Department of medical psychology, VU University Medical Center, Amsterdam, Netherlands; 3 Department of pediatrics, division of oncology-hematology, Radboud University, Nijmegen, Netherlands; 4 Department of pediatric immunology, hematology, oncology, bone marrow transplant and auto-immune diseases, Leiden University Medical Center, Leiden, Netherlands; 5 Department of pediatrics, division of oncology-hematology. VU University Medical Center, Amsterdam, Netherlands

## Abstract

**Background:**

With the increase of pediatric cancer survival rates, late effects and quality of life (QoL) have received more attention. Disturbed sleep in pediatric cancer is a common clinical observation, but research on this subject is sparse. In general, sleep problems can lead to significant morbidity and are associated with impaired QoL. Information on sleep is essential to develop interventions to improve QoL.

**Methods:**

Children (2-18 years) with acute lymphoblastic leukemia (ALL) were eligible for this multi-center study. The Children's Sleep Habits Questionnaire (CSHQ), Child Health Questionnaire (CHQ) and Pediatric Quality of Life Inventory 3.0™ Acute Cancer Version (PedsQL) were used to assess sleep and QoL halfway through maintenance therapy. Sleep and QoL were measured during and after dexamethasone treatment (on-dex and off-dex).

**Results:**

Seventeen children participated (age 6.7 ± 3.3 years, 44% boys). Children with ALL had more sleep problems and a lower QoL compared to the norm. There were no differences on-dex and off-dex. Pain (r = -0.6; p = 0.029) and worry (r = -0.5; p = 0.034) showed a moderate negative association with sleep. Reduced overall QoL was moderately associated with impaired overall sleep (r = -0.6; p = 0.014) and more problems with sleep anxiety (r = -0.8; p = 0.003), sleep onset delay (r = -0.5; p = 0.037), daytime sleepiness (r = -0.5; p = 0.044) and night wakenings (r = -0.6; p = 0.017).

**Conclusion:**

QoL is impaired in children during cancer treatment. The results of this study suggest that impaired sleep may be a contributing determinant. Consequently, enhanced counseling and treatment of sleep problems might improve QoL. It is important to conduct more extensive studies to confirm these findings and provide more detailed information on the relationship between sleep and QoL, and on factors affecting sleep in pediatric ALL and in children with cancer in general.

## Background

Survival rates for childhood cancer are increasing, especially for the most common type of pediatric cancer, acute lymphoblastic leukemia (ALL). Over the past decades survival for ALL has reached 80-85% [1]. The improved survival rates have led to more attention to other outcomes, such as quality of life (QoL), fatigue and to a lesser extent, sleep. In clinical practice it seems that sleep related problems are not uncommon during ALL treatment, but research on this subject is sparse.

Sleep disorders in children can lead to significant behavioral and cognitive morbidities. The prevalence of sleep problems in children in the general population is up to 30% [2,3]. Gender and age influence sleep [3-5], and some sleep problems are more common during certain stages of child development, such as night wakings during infancy [6] and sleep onset delay in older children [3]. Children with sleep difficulties experience higher rates of behavioral problems, depression, anxiety in adulthood, and impaired cognitive function and emotional development [6-11]. Sleep problems are more common in certain medical conditions, such as chronic pain, attention deficit hyperactivity disorder, and autism [12-14]. Information on sleep in cancer patients is limited. Reported prevalence of sleep problems in adult cancer patients varies greatly but seems higher than in healthy people [15,16]. Mulrooney et al.[17] reported on sleep in a large pediatric cancer survivor cohort using a sleep questionnaire, and found a lower sleep quality compared to siblings, although the authors argue that the differences might not be clinically important. During ALL treatment children seem to experience more sleep problems, and the use of corticosteroids negatively affects sleep [18,19]. Hinds et al.[4] performed actigraphy in children with ALL and found that dexamethasone alters sleep. During dexamethasone treatment duration of sleep was increased and there was an increase in nighttime awakenings, restless sleep and nap time.

An association between poor quality of sleep and impaired health-related quality of life and well being has been found in several populations, such as children with chronic pain and survivors of childhood cancer [13,17,20,21]. To our knowledge, the relationship between sleep and QoL during ALL treatment has not yet been studied. Insight in the relationship between sleep and QoL may help develop interventions in order to improve QoL during and after childhood ALL treatment. Therefore the main objective of this study was to assess sleep, QoL, and the relationship between sleep and QoL, in children during maintenance treatment for ALL. We hypothesized that impaired sleep is associated with impaired QoL, and that sleep and QoL are negatively affected by dexamethasone.

## Methods

### Patients

Eligible patients were between two and eighteen years of age, and were receiving ALL maintenance therapy according to the Dutch Childhood Oncology Group ALL10 medium risk protocol at one of the three participating tertiary care hospitals (VU University Medical Center, Amsterdam; Leiden University Medical Center, Leiden; St Radboud University Medical Center, Nijmegen). Children were recruited from August 2006 till October 2007 at the VU University Medical Center Amsterdam, at the Leiden University Medical Center from February till August 2007, and at the Radboud University Medical Center Nijmegen from January till July 2007. Eligibility was restricted to one risk group in order to keep treatment variables similar, and the medium-risk (MR) group was chosen because it is the largest category. Participants had to be Dutch speaking and provide informed consent. Children with pre-existent serious morbidity that was thought to influence sleep and QoL, such as a psychiatric or neurological disorder, were excluded. The study was approved by the institutional review boards.

Sleep was assessed halfway through maintenance therapy. Because the MR maintenance protocol includes cyclic corticosteroids (6 mg/m2 dexamethasone per day, every three weeks for five consecutive days), measurements were done twice to assess the influence of dexamethasone: once at the end of a dexamethasone period (on-dex) and the second time at the end of a dexamethasone free period (off-dex) five weeks later. Questionnaires were sent to the participant's home with instructions and a stamped return envelop. The sample size was based on QoL differences on-dex and off-dex as found before in Dutch children with ALL [22]. Using mean and SD scores of the physical summary score of the Child Health Questionnaire, a sample size of 19 was required in order to have 80% power to detect an effect size of 0.6 at a 5% significance level (one sided test).

### Questionnaires

The Children's Sleep Habits Questionnaire (CSHQ) is a one-week recall, 33 item parental questionnaire that was developed as a sleep screening tool for school-aged children and has been shown to be a useful screening tool in younger children as well [23,24]. Both the original and the Dutch version of the CSHQ have adequate psychometric properties [23,25]. The frequency of sleep behavior is rated for the most recent "typical" week on a three point Likert scale, with the response options *usually *(5 to 7 times per week), *sometimes *(2 to 4 times per week) and *rarely *(0 to 1 time per week). A higher score indicates more sleep disturbances. Information on habitual bedtime, morning wake-up time and sleep duration was collected additionally. The CSHQ allows for a total score over 33 items and subscales scores on a number of key sleep domains: bedtime resistance (6 items), sleep-onset delay (1 item), sleep duration (3 items), sleep anxiety (4 items), night wakening (3 items), parasomnias (7 items), sleep-disordered breathing (3 items) and daytime sleepiness (8 items).

The Dutch version of the Child Health Questionnaire 50 items parent form (CHQ) is a generic QoL assessment tool and has shown good reliability and validity [26,27]. The CHQ has been used in several pediatric oncology studies [22,28,29]. This instrument covers the physical, emotional and social well-being of children and allows for two summary scores (physical and psychosocial). Items are scored using a four to six point Likert scale and converted to a 0 to 100 point continuum, with higher scores indicating better QoL. The original reference period of the CHQ (four weeks) was adjusted to suit the CSHQ recall period (one week). Dutch population norms are available and allow for a comparison with the Dutch healthy population [27]. Certain questions, i.e. "My child seems to be less healthy than other children I know" were felt not to be appropriate during ALL maintenance treatment because of the repetitive setup of the assessments. For these questions (number 1 and 8), mean scores as found in a previous study in Dutch children halfway ALL maintenance were imputed [22]. The CHQ was designed for children five years and up. Although the Infant and Toddler Quality of Life Questionnaire would have been more appropriate for the few younger children (n = 3) in our study sample [30], at the time of the design of our study, no validated Dutch version and norms were available.

The Pediatric Quality of Life Inventory 3.0™ Acute Cancer Version (PedsQL) is a reliable and valid cancer specific questionnaire [31]. It has frequently been used in pediatric oncology studies [22,32-34] and includes subscales with age-specific questions for determining problems in relevant areas during cancer treatment such as pain, nausea, treatment and procedural anxiety, worry, cognitive problems, perceived physical appearance and communication. Items are scored using a four point Likert scale and reflect on the past week. Higher scores indicate better QoL.

### Analysis

The Statistical Package for Social Sciences for Macintosh version 18.0 was used for all data analyses. For the description of demographic variables and questionnaire scores, medium and inter quartile range (IQR), and mean and standard deviation (SD) scores were calculated. To allow for age-specific differences in sleep, three groups were identified: <5 years, 5-7 years, and >7 years. Differences between Dutch CSHQ norm scores and ALL scores were assessed using Mann-Whitney U tests. CHQ differences with Dutch population norms were calculated using one-sample t-tests. On and off dexamethasone scores were assessed using Wilcoxon signed ranks tests. Correlations between QoL and sleep were calculated using Spearman's correlations. For this purpose individual sleep scores were corrected for age-specific norms. Correlations between 0.2 and <0.5 were considered small, between ≥0.5 and <0.8 moderate, and ≥0.8 were considered strong. Moderate or strong significant correlations were considered to potentially be clinically relevant and are reported in this study. Significance level was set at two-sided p < 0.05 for all analyses.

## Results

### Demographics

Twenty-one children and their parents were eligible and were invited to participate. Nineteen provided written informed consent, one parent thought the study burden was too high and declined participation, reasons for not participating are unknown for the another child. No demographic information was available on these children. Questionnaires were not returned for one child (a 10 year old male), and one questionnaire was not filled out completely, so in total seventeen children could be analyzed. Mean age at diagnosis was 6.7 years (SD 3.3), 44% were boys.

### Sleep

There appeared to be more sleep problems in children with ALL compared to healthy children. Significant differences were found for bedtime resistance (p = 0.020), sleep anxiety (p = 0.016) and night wakening (p = 0.024). Children with ALL had fewer problems with sleep onset delay (p = 0.024). In the youngest age group (under five years, n = 6) those with ALL scored significantly higher on the CSHQ total score (p = 0.034), and also had more problems with sleep anxiety (p = 0.003), night wakening (p = 0.047) and parasomnias (p = 0.037). In the middle age group (five to seven years, n = 6) children with ALL scored significantly higher for bedtime resistance (p = 0.025). There were no significant differences in the oldest age group (n = 5). Results are shown in table 1. Sleep did not differ between on-dex and off-dex measurements, except for the sleep onset delay subscale for which the off-dex score was significantly higher, indicating more problems (p = 0.02).

**Table 1:** Children's Sleep Habits Questionnaire scores (median and inter quartile range)

	All			<5 years			5-7 years			>7 years		
	
CSHQ score	ALL (n = 17)	Norm* (n = 1507)	p	ALL (n = 6)	Norm* (n = 174)	p	ALL (n = 6)	Norm* (n = 315)	p	ALL (n = 5)	Norm* (n = 1018)	p
Total score	41.00 (11.50)	39.00 (6.02)	.076	45.00 (14.00)	40.00 (8.00)	.034	40.00 (13.00)	39.00 (6.00)	.786	41.00 (14.00)	39.00 (7.00)	.780
Subscale item												
Bedtime resistance	6.38 (5.00)	6.00 (1.00)	.020	8.69 (6.00)	6.00 (1.00)	.068	8.50 (5.75)	6.00 (1.00)	.025	6.0 (0.50)	6.00 (1.00)	.526
Sleep onset delay	1.00 (0.00)	1.00 (0.00)	.024	1.00 (0.00)	1.00 (0.00)	.354	1.00 (0.00)	1.00 (0.00)	.274	1.00 (0.00)	1.00 (1.00)	.174
Sleep duration	3.00 (0.00)	3.00 (1.00)	.343	3.00 (0.25)	3.00 (1.00)	.499	3.00 (1.00)	3.00 (1.00)	.736	3.00 (2.00)	3.00 (1.00)	.739
Sleep anxiety	5.00 (3.75)	4.00 (1.00)	.016	8.00 (2.50)	5.00 (2.00)	.003	5.00 (2.50)	4.23 (2.00)	.484	4.00 (1.50)	4.00 (1.00)	.965
Night wakening	4.00 (2.00)	3.00 (1.00)	.024	5.00 (4.25)	3.18 (2.00)	.047	3.00 (2.00)	3.00 (1.00)	.776	4.00 (2.50)	3.00 (1.00)	.198
Parasomnias	9.00 (3.00)	8.00 (2.21)	.500	10.14 (1.86)	9.00 (3.00)	.037	8.00 (2.25)	8.00 (3.00)	.498	7.00 (1.50)	8.00 (2.00)	.224
Sleep disordered breathing	3.00 (0.00)	3.00 (0.00)	.275	3.00 (1.00)	3.00 (1.00)	.983	3.00 (0.00)	3.00 (0.04)	.161	3.00 (0.00)	3.00 (0.00)	.270
Daytime sleepiness	11.00 (5.00)	11.00 (4.00)	.223	11.50 (5.00)	10.11 (3.00)	.211	10.50 (5.75)	10.65 (3.00)	.775	13.0 (7.00)	11.00 (4.00)	.156

Scores are represented for all ALL children, the Dutch reference population and per age group. Higher scores indicate more sleep problems. Scores were calculated if <50% of responses were missing. N = number of children included. * Reference population consisting of healthy school-aged Dutch children [3].

In the youngest age group, children with ALL had a median sleep duration that was 30 minutes longer than the sleep duration in healthy children; this was a significant difference (p = 0.042). There were no other differences in sleep times. Sleep times on-dex and off-dex were not significantly different.

### Quality of Life

QoL (both on-dex and off-dex) was lower in ALL compared to Dutch CHQ population norms. This was significant for all scales except for family cohesion and off-dex mental health. See table 2. There were no statistically significant differences in QoL measured with the CHQ and the PedsQL between on-dex and off-dex scores.

**Table 2:** Child Health Questionnaire mean (SD) scores

	Dutch norm	On-dex	Norm versus on-dex p	Off-dex	Norm versus off-dex p
Physical Functioning	99.3 (4.3)	60.5 (26.2)	<0.001	66.3 (26.3)	<0.001
Role Limitations: emotional/behaviour	97.9 (13.9)	83.3 (26.2)	0.031	87.4 (13.8)	0.011
Role Limitations: physical	95.8 (15.6)	62.0 (33.2)	<0.001	65.6 (37.0)	0.007
Bodily Pain	85.7 (17.2)	66.7 (32.4)	0.023	73.1 (20.2)	0.025
General Behaviour	78.5 (13.1)	69.6 (16.9)	0.040	70.6 (14.2)	0.041
Mental Health	81.4 (12.1)	72.2 (16.3)	0.029	74.4 (14.4)	0.069
Self-esteem	79.2 (11.0)	70.0 (13.3)	0.005	67.2 (19.5)	0.032
Parental Impact: emotional	86.3 (15.2)	74.1 (18.3)	0.011	71.9 (20.2)	0.012
Parental Impact: time	94.0 (13.0)	64.8 (26.7)	<0.001	64.6 (25.4)	<0.001
Family Activities	91.5 (11.9)	69.2 (19.7)	<0.001	70.3 (20.4)	0.001
Family Cohesion	72.2 (19.4)	63.3 (19.2)	0.066	63.1 (21.0)	0.105
Physical Summary Score Z-score*	56.4 (5.7)	33.4 (13.4)	<0.001	37.9 (12.2)	<0.001
Psychosocial Summary Score Z-score*	53.2 (6.4)	48.5 (8.9)	0.040	48.8 (7.5)	0.046

Higher scores indicate a better QoL. There were no significant differences in on-dex and off-dex scores. Dutch norm scores consist of a sample of healthy school-aged children [27]. Imputed mean general health subscale scores (based on a previous study [22], see methods): on-dex 47.5 and off-dex 50.0.

* Physical and Psychosocial CHQ summary scores based on a factor-analytical model on U.S. population samples. A score of 50 represents the mean in the general U.S. population.

### Sleep and Quality of Life

On-dex, the CHQ overall physical QoL was negatively correlated with overall sleep (r = -0.6; p = 0.014), sleep anxiety (r = -0.6; p = 0.021) and night wakenings (r = -0.6; p = 0.017). Psychosocial QoL negatively correlated with daytime sleepiness (r = -0.5; p = 0.044) and sleep onset delay (r = -0.5; p = 0.046). Off-dex, psychosocial QoL was negatively correlated with sleep anxiety (r = -0.8; p = 0.003); pain was negatively correlated with overall sleep (r = -0.6; p = 0.029) and daytime sleepiness (r = -0.6; p = 0.027). The subscale family activities was negatively correlated with sleep onset delay (r = -0.5; p = 0.039).

Regarding the PedsQL during the on-dex measurement, worry was negatively correlated with overall sleep (r = -0.5; p = 0.034), overall QoL was negatively correlated with daytime sleepiness (r = -0.5; p = 0.037). Parasomnias were negatively correlated with procedure anxiety (r = -0.5; p = 0.03), treatment anxiety (r = -0.5; p = 0.03), and cognitive functioning (r = -0.5; p = 0.03). Sleep anxiety was negatively correlated with worry (r = -0.7; p = 0.004) and nausea (r = -0.6; p = 0.009). Sleep duration was negatively correlated with cognition (r = -0.5; p = 0.032), daytime sleepiness was negatively correlated with physical appearance (r = -0.5; p = 0.028). Sleep onset delay was negatively correlated with procedure anxiety (r = -0.6; p = 0.013). Off-dex daytime sleepiness was associated with cognitive functioning (r = -0.6; p = 0.024) and physical appearance (r = -0.5, p = 0.036).

## Discussion

This study shows that sleep is affected in children during ALL maintenance compared to healthy children, with the largest differences in the younger age groups. Bedtime resistance, sleep anxiety, night wakening, and parasomnias were impaired, but children with ALL had fewer problems with sleep onset delay. Sleep duration was significantly longer in the youngest children with ALL compared with their healthy peers. Previous studies in pediatric ALL also found impaired sleep and increased sleep duration during corticosteroid treatment [4,18] but most have not correlated these results with QoL and have not used a validated generic sleep questionnaire for children. Generic sleep questionnaires can provide uniform, detailed and comparable information regarding specific sleep problems compared to sleep diaries and actigraphy.

QoL was impaired compared to healthy children, which is consistent with previous research [22]. In contrast to other studies however, no differences were found in sleep and QoL on-dex and off-dex [4,22,35,36]. Although this study was powered on QoL differences on-dex and off-dex as found before in Dutch children with ALL [22], the corticosteroid regimen was different in the previous study (i.e. 14 days of dexamethasone in a 7 week cycle as compared to 5 days of dexamethasone in a 3 week schedule in the current study). The shorter corticosteroid cycle in the current study may have led to smaller on-dex and off-dex differences, potentially explaining the absence of statistically significant differences.

Sleep and QoL were negatively correlated on many items. Most correlations were moderate, with Spearman's rho between 0.5 and 0.8. In our study the QoL item pain was negatively associated with overall sleep and daytime sleepiness, which is consistent with previous research on the influence of pain on sleep [13,21]. Anxiety and stress have been described to influence sleep [16,37], which corresponds to our study in which worry and treatment/procedure anxiety were negatively associated with overall sleep, sleep anxiety, parasomnias and sleep onset delay. Reduced overall QoL was associated with impaired overall sleep and more problems with sleep anxiety, sleep onset delay, daytime sleepiness and night wakenings. Similar results have been found in children with chronic pain [13] and children referred to a sleep disorder clinic [20], but was not yet demonstrated in children with ALL.

This is an exploratory, cross-sectional, study and it has several limitations. Therefore, results should be interpreted with care. Besides the cross-sectional character of the study, the number of patients is small. The required sample size was not completely reached so a lack of power could have contributed to the absence of significant differences in QoL on-dex and off-dex. Further, both sleep and QoL were measured using parental reports because most children were too young for self reports. In QoL it is well known that children and parents do not always agree [38], and similar results have been found in sleep studies [37]. Finally, although the assessment of child sleep by parental questionnaire has shown adequate correlation with objective sleep measures such as actigraphy for sleep schedules, parents are less accurate in assessing sleep quality [39-41]. Nevertheless, this study will provide a basis for further research with more robust analysis on this interesting topic. In future research, we suggest including other variables that might influence sleep, such as depression [10], pain [13], hospitalization [42], and treatment regimens such as corticosteroids and irradiation enabling a more comprehensive analysis. Objective sleep measures as well as subjective self reports should be included whenever possible.

## Conclusion

The success of advancement in pediatric oncology has lead to a decrease in mortality and an increased attention for the burden of treatment for both the patient and family. QoL is impaired in children during cancer treatment, and the results of this study suggest that impaired sleep might be one of the contributing factors. Better counseling and treatment of sleep problems might improve QoL. It is therefore important to conduct more extensive studies to confirm these findings and provide more detailed information on the relationship between sleep and QoL, and on factors affecting sleep in pediatric ALL and in children with cancer in general.

## Abbreviations

ALL: Acute lymphoblastic leukemia; CHQ: Child Health Questionnaire; CSHQ: Children's Sleep Habits Questionnaire; IQR: Inter quartile range; PedsQL: Pediatric Quality of Life Inventory 3.0™ Acute Cancer Version; QoL: Quality of life; SD - Standard deviation.

## Competing interests

The authors declare that they have no competing interests.

## Authors' contributions

RVL conceived of and designed the study, coordinated the study and acquired data, performed the statistical analysis and drafted the manuscript. JHU, GJK and RJG helped to design the study, made contributions to the interpretation of data and were involved in the drafting and critical revision of the manuscript. RME and PHO helped with the acquisition of data and critically revised the manuscript. All authors have given final approval of the version to be published.
